# Multicenter study of lumbar discectomy with Barricaid annular closure device for prevention of lumbar disc reherniation in US patients: A historically controlled post-market study protocol

**DOI:** 10.1097/MD.0000000000016953

**Published:** 2019-08-30

**Authors:** K. Brandon Strenge, Christian P. DiPaola, Larry E. Miller, Clint P. Hill, Robert G. Whitmore

**Affiliations:** a The Orthopaedic Institute, Paducah, Kentucky; b Department of Orthopaedics and Rehabilitation, UMass Memorial Medical Center, Worcester; c Miller Scientific Consulting, Inc., Asheville, North Carolina; d Department of Neurosurgery, Lahey Hospital and Medical Center, Burlington, Massachusetts.

**Keywords:** annular closure device, Barricaid, lumbar discectomy, lumbar herniation, sciatica

## Abstract

**Background:**

Patients with large defects in the annulus fibrosus following lumbar discectomy have high rates of symptomatic reherniation. The Barricaid annular closure device provides durable occlusion of the annular defect and has been shown to significantly lower the risk of symptomatic reherniation in a large European randomized trial. However, the performance of the Barricaid device in a United States (US) population has not been previously reported.

**Design and methods:**

This is a historically controlled post-market multicenter study to determine the safety and efficacy of the Barricaid device when used in addition to primary lumbar discectomy in a US population. A total of 75 patients with large annular defects will receive the Barricaid device following lumbar discectomy at up to 25 sites in the US and will return for clinical and imaging follow-up at 4 weeks, 3 months, and 1 year. Trial oversight will be provided by a data safety monitoring board and imaging studies will be read by an independent imaging core laboratory. Patients treated with the Barricaid device in a previous European randomized trial with comparable eligibility criteria, surgical procedures, and outcome measures will serve as historical controls. Main outcomes will include back pain severity, leg pain severity, Oswestry Disability Index, health utility on the EuroQol-5 Dimension questionnaire, complications, symptomatic reherniation, and reoperation. Propensity score adjustment using inverse probability of treatment weighting will be used to adjust for differences in baseline patient characteristics between the US trial participants and European historical controls.

**Ethics and dissemination:**

This study was approved by a central institutional review board. The study results of this trial will be widely disseminated at conference proceedings and published in peer-reviewed journals. The outcomes of this study will have important clinical and economic implications for all stakeholders involved in treating patients with lumbar discectomy in the US.

**Study registration:**

ClinicalTrials.gov (https://clinicaltrials.gov): NCT03986580.

**Level of evidence:**

3.

## 1 Introduction

Lumbar disc herniation is a localized displacement of disc material beyond the normal margins of the intervertebral disc space^[1]^ and is the primary cause of sciatica, which affects between 1% and 5% of the adult population each year.^[2]^ First-line treatments for sciatica are nonsurgical and may consist of physical therapy, pharmacologic therapy, and/or epidural steroid injection. Regardless of whether conservative treatment is undertaken, acute sciatica symptoms eventually subside in most patients.^[3,4]^ In patients whose symptoms are resistant to conservative treatments, lumbar discectomy may be considered. Surgery typically results in faster symptom relief than continued conservative care^[5]^ and this clinical advantage persists over longer term follow-up.^[1]^ Consequently, lumbar discectomy has emerged as one of the most commonly performed spinal surgeries.^[6]^

Among patients undergoing lumbar discectomy, recurrent disc herniation occurs in 7% to 18% of patients within 2 years.^[7–10]^ Symptomatic reherniation requires reoperation in nearly 80% of cases.^[11]^ Patients at highest risk for reherniation are those with an annular defect of at least 6 mm width at completion of the lumbar discectomy procedure.^[12]^ This high-risk patient population is easily identifiable by measuring the annular defect width with a Penfield probe or specialized measurement tools. Historically, there has been a considerable unmet therapeutic need in this high-risk patient population. Over the last several decades, multiple attempts at the development of techniques and technologies to repair annular defects have been met with limited success.^[13]^

A bone-anchored device intended to occlude large annular defects after lumbar discectomy (Barricaid, Intrinsic Therapeutics, Woburn, MA) received Food and Drug Administration approval in 2019 based, in part, on 2-year safety and efficacy results from a 554-patient randomized trial conducted in Europe.^[14]^ Due to potential regional differences in patient characteristics or surgical practices, it is important to confirm the generalizability of these results to patients in the United States (US). The purpose of this historically controlled post-market confirmatory study is to report the safety and efficacy of the Barricaid annular closure device when used in addition to a primary lumbar discectomy procedure in a US patient population.

## 2 Design and methods

The protocol for this study was developed in accordance with the SPIRIT 2013 guidance for protocols of clinical trials.^[15]^

### 2.1 Study design

This is a prospective, multicenter, single-arm, historically controlled, confirmatory study that will be performed at up to 25 sites in the US. Patient recruitment is planned to begin in February 2020. The total study duration is expected to be 1.5 years, with 6 months of patient recruitment and 1 year of follow-up. Patient outcomes in this US trial of the Barricaid device (US Barricaid) will be descriptively compared with those from the Barricaid arm of a European randomized controlled trial consisting of 276 patients (EU Barricaid Controls).^[14]^ Propensity score methods will be used to adjust for differences in baseline patient characteristics between the US Barricaid patients and EU Barricaid Controls. The trial was prospectively registered at ClinicalTrials.gov (NCT03986580) before first patient enrollment. Trial oversight will be provided by a data safety monitoring board and data will be routinely monitored for accuracy.

### 2.2 Participants and eligibility criteria

Participants will undergo preoperative axial and sagittal magnetic resonance imaging (MRI) of the lumbar spine, low-dose multiplanar computed tomography of the target lumbar level, and 4-view x-rays (standing flexion-extension, lateral, anteroposterior). Key eligibility criteria are lumbar disc herniation at L4–L5 or L5–S1, leg pain severity at least 40 mm on a visual analogue scale of 100 mm length, a score of at least 40 on the Oswestry Disability Index (ODI)^[16]^ despite at least 6 weeks of nonsurgical management, and a positive straight leg raise sign on physical examination. Key exclusion criteria are previous spinal surgery at the herniated level, spondylolisthesis with at least 25% slip at the index level, and lumbar osteoporosis. Patients who meet all preoperative eligibility criteria will undergo limited lumbar discectomy during which the final eligibility criterion regarding annular defect size will be evaluated. A complete list of study eligibility criteria is provided in Table 1.

**Table 1:**
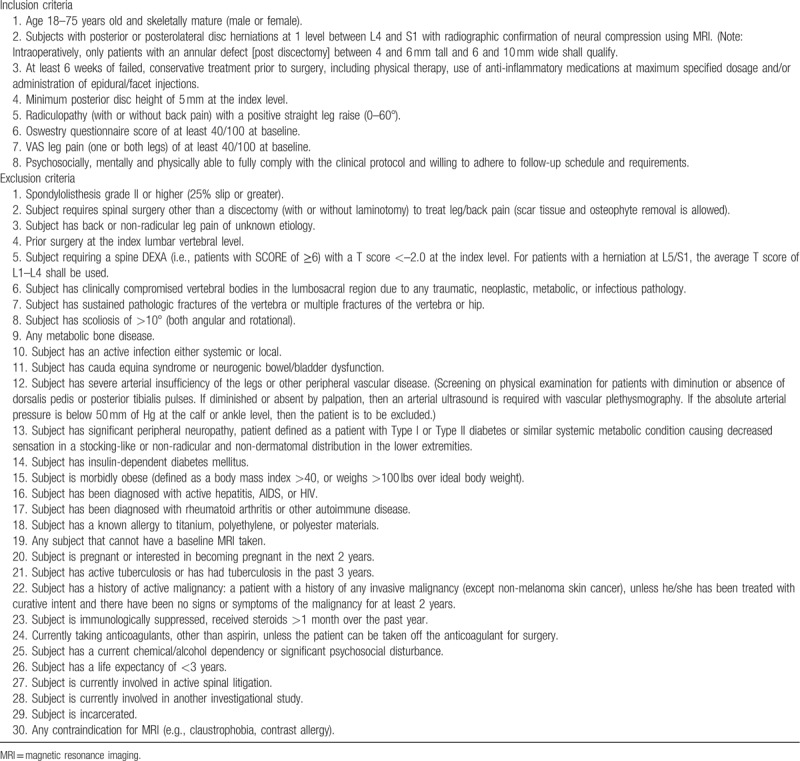
Patient eligibility criteria.

### 2.3 Surgical procedure

Patients will be treated with limited lumbar microdiscectomy using an interlaminar transflaval approach as described by Spengler.^[17]^ At completion of the limited discectomy procedure, patients will then be assessed for the final eligibility criterion by measuring the size of the annular defect. Patients with a large annular defect, defined as 4 to 6 mm tall and 6 to 10 mm wide, will be enrolled in the trial and treated with the Barricaid device. Patients with annular defect sizes outside of the eligible range will be excluded from further trial participation.

**Figure 1:**
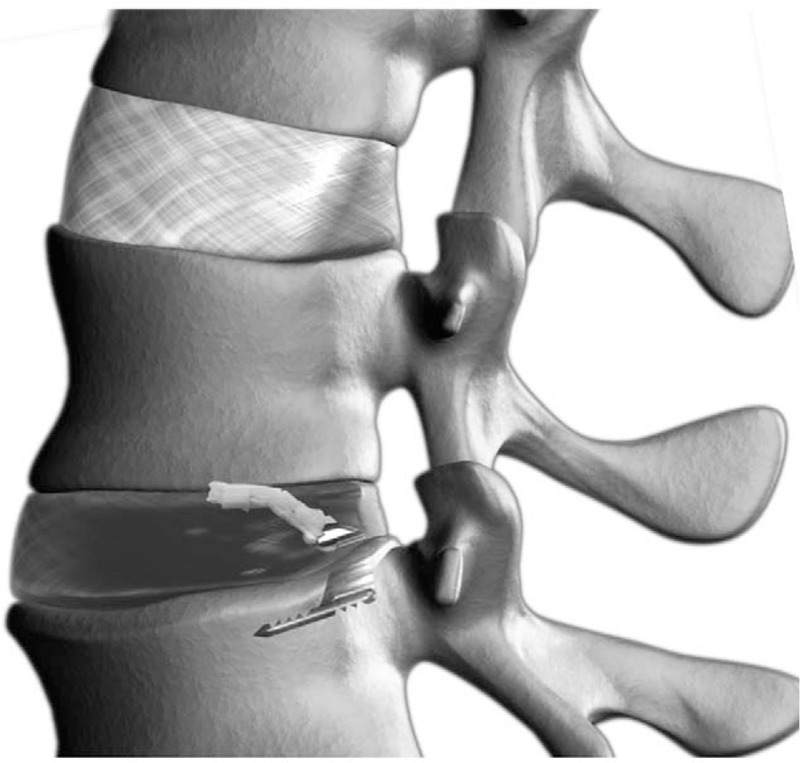
The Barricaid annular closure device. The device is comprised of a flexible polymer occlusion component that is attached to a titanium bone anchor.

The Barricaid device is a permanent implant that has 2 major subcomponents: a flexible woven polymer fabric component intended to close the annular defect, and a bone anchor to secure the device to an adjacent vertebral body (Fig. 1). The occlusion component consists of a flexible polymer that is designed to prevent reherniation by physically blocking the annulus at the post-surgery defect to maintain hydrostatic pressure inside the nucleus pulposus,^[18]^ and containing a platinum-iridium radiopaque marker to permit radiographic visualization. The anchor component is a saw-toothed titanium alloy that is secured into either the caudal-adjacent or cranial-adjacent vertebral body to resist migration.

After confirmation of a large annular defect, an alignment trial will be performed under fluoroscopic control to establish the correct position and angle of the Barricaid device. Next, the device will be implanted under fluoroscopic guidance by impacting the anchor into the vertebral body while the occlusion component simultaneously enters the annular defect to prevent expulsion of disc material into the extradiscal space. After fluoroscopic confirmation of correct device placement, the surgical site will be inspected and standard wound closure will be performed.

### 2.4 Outcomes

Follow-up visits will occur at 4 weeks, 3 months, and 1 year posttreatment. Magnetic resonance imaging with axial and sagittal images of the lumbar spine, low-dose multiplanar computed tomography of the target level only, and x-rays will be performed during follow-up. Back pain severity, leg pain severity, ODI, EuroQol-5 Dimension (EQ-5D) questionnaire, complications, symptomatic reherniation, and reoperation will be assessed at each follow-up visit. A schedule of patient assessments during the study is provided in Table 2.

**Table 2:**
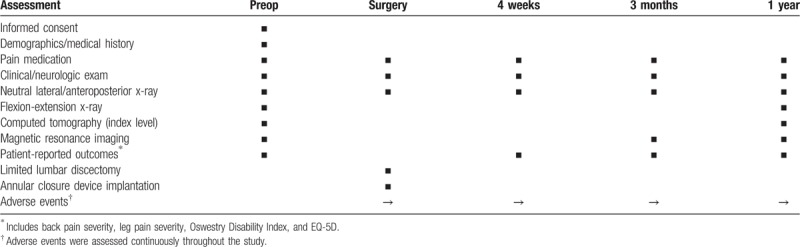
Study assessments at each follow-up interval.

**Figure 2:**
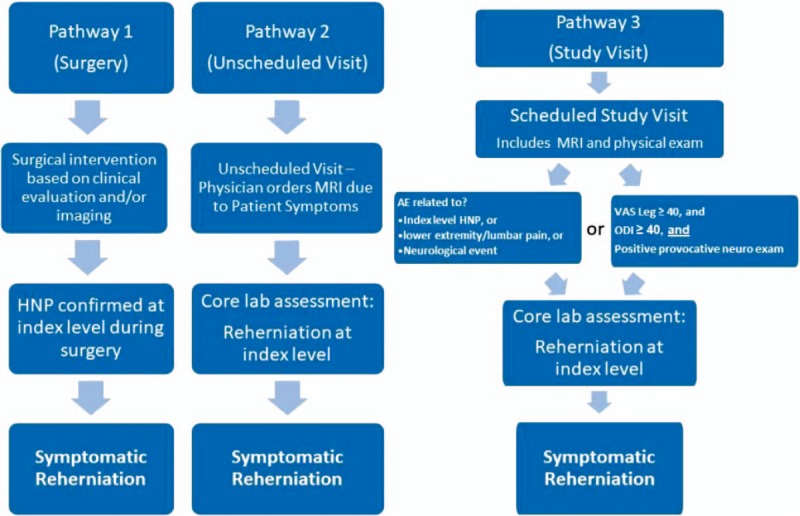
Algorithm to determine occurrence of symptomatic reherniation. AE = adverse event, HNP = herniated nucleus pulposus, MRI = magnetic resonance imaging, ODI = Oswestry Disability Index, VAS = visual analogue scale.

Back and leg pain severity will be separately assessed on a 100 mm visual analogue scale. Back disability will be evaluated with the ODI using a 0 to 100 scale where higher scores represent greater disability. Health utility will be evaluated with the EQ-5D questionnaire.^[19]^ This patient-reported questionnaire consists of 5 domains (mobility, self-care, usual activities, pain/discomfort, and anxiety/depression), each of which can take 1 of 3 responses. Possible responses include 3 levels of severity (no problems/some or moderate problems/extreme problems) within each EQ-5D dimension. The occurrence of adverse events will be evaluated at each visit and adjudicated for seriousness and relation to the procedure or device by an independent data safety monitoring board. Symptomatic reherniation will be defined as: confirmed reherniation during a reoperation, imaging core lab confirmation of reherniation based on MRI performed at an unscheduled visit due to patient symptoms, or imaging core lab confirmation of reherniation based on MRI performed at an scheduled visit in patients with ODI ≥40 and positive leg raise sign, or with an adverse event deemed related to reherniation, lumbar/leg pain, or a neurological event (Fig. 2). Index-level reoperations will be defined as any surgical procedure performed at the level of the original herniation, regardless of side or reason, during follow-up.

An independent imaging core laboratory will read imaging to assess device status and anatomical characteristics following the procedure. Key imaging variables will include Modic changes, Pfirrman grade, anterior and posterior ossification, spontaneous fusion, disc degeneration, herniation recurrence, annular tears/fissures, and device events such as device integrity, migration, and subsidence.

The primary endpoint of the study is the incidence of symptomatic reherniation through the 3-month follow-up visit. Secondary endpoints of the study will include symptomatic reherniation, reoperation, back pain severity, leg pain severity, ODI, and adverse events at 3 months and 1-year follow-up.

### 2.5 Propensity score adjustment

We anticipate that patients enrolled in the US Barricaid trial may differ from the EU Barricaid Controls with respect to certain baseline characteristics. Therefore, we will prospectively collect clinically relevant baseline variables including age, sex, body mass index, smoking history, level of herniation, average disc height, herniation type, back pain severity, leg pain severity, and ODI. We will also obtain patient-level data from the EU Barricaid Controls for these variables from the study sponsor. We will then calculate propensity scores using binomial logistic regression to estimate the probability that patients would be enrolled in the US Barricaid trial. Propensity score adjustment using inverse probability of treatment weighting (IPTW) will be used to control for potential baseline covariate imbalances between study groups, thereby providing unbiased estimates of average treatment effects. Compared with other methods of propensity score adjustment such as stratification, matching, or covariate adjustment, IPTW is preferable for the analysis of differences in proportions,^[20]^ which coincides with the planned primary endpoint analysis of this trial. We will verify the performance of the propensity score model by comparing the distribution of covariates between study groups before and after propensity score adjustment using the absolute average standardized difference (ASD) statistic. The ASD will be calculated as the difference in means or proportions between study groups divided by the pooled standard deviation. A negligible difference between treatment groups will be defined as an ASD of <0.1.^[21]^

### 2.6 Statistical analysis

Using a Clopper-Pearson exact test, a sample size of 75 patients (70 evaluable at 3 months) produces a 2-sided 95% confidence interval ranging from 0.4% to 10.2% when the symptomatic reherniation rate is 3.0%. The primary endpoint will be calculated as the number of patients who experience symptomatic reherniation through the end of the 3-month follow-up visit (postoperative day 105) divided by the sum of patients who return for the 3-month follow-up visit and all patients with confirmed symptomatic reherniation who fail to return for this visit. The propensity score-adjusted primary endpoint results will be descriptively reported for patients treated with the Barricaid device in each study, with no formal hypothesis testing. The difference in propensity score-adjusted 3-month reherniation risk between the US Barricaid trial participants and the EU Barricaid Controls will be reported as an absolute risk difference and 95% confidence interval.

Primary study results will be derived from propensity score-adjusted comparisons. Group comparisons without propensity-score adjustment will be performed as a sensitivity analysis. Baseline characteristics will be summarized with standard descriptive statistics. Categorical variables will be described with percents and counts. Continuous variables will be described with means and standard deviations, or medians and ranges. Longitudinal changes in patient-reported outcomes will be evaluated with a mixed model analysis of variance. Categorical efficacy data will be evaluated as the number of patients with an event divided by the number of patients with follow-up data plus the number of patients with an event who miss the follow-up visit. Symptomatic reherniation and reoperation rates will be evaluated in a sensitivity analysis using Kaplan–Meier time-to-event methods. Missing data imputation will not be performed.

### 2.7 Ethics and dissemination

The protocol for this clinical trial was approved by Western Institutional Review Board (Puyallup, WA) and all enrolled subjects will provide informed consent before study participation. The study results will be widely disseminated at conference presentations and published in peer-reviewed journals.

## 3 Discussion

Annular healing following lumbar discectomy occurs slowly, if at all, and ultimately yields biomechanically inferior fibrous tissue with reduced capacity to accommodate tensile force.^[22–24]^ This makes reherniation of disc material likely to occur under lower biomechanical stresses, especially in patients with large post-surgical annular defects. Several strategies to repair, replace, or regenerate the damaged annulus have been evaluated yet none have resulted in a clinically proven strategy to generate appropriate healing and prevent reherniation. It is likely that prior attempts at annulus repair have failed because exterior repairs are not matched to the demands of intradiscal tensile forces.^[25]^ Since the Barricaid device is anchored into an adjacent vertebral body, this may provide for a more durable repair.

The primary goal of this prospective post-market study of the Barricaid annular closure device is to determine whether clinical outcomes with this device in the US are comparable to those observed in a previous randomized trial with the same device in Europe.^[14]^ The design of this study and the conclusions to be drawn will be strengthened by the fact that patient eligibility criteria, surgical technique, device characteristics, and follow-up methodology including imaging and outcome reporting are comparable in the US Barricaid Trial and the EU Barricaid Controls. Further, potential group differences in important patient characteristics will be accounted for using propensity score methods. Thus, this observational study was appropriately designed to control for several important design factors that might have otherwise introduced bias into the results.

The primary endpoint of this study will be evaluated at 3 months and patients will continue in follow-up for 1 year after surgery. Although it could be argued that longer follow-up is needed in this relatively young patient population, the 3-month primary endpoint analysis is justified since early clinical outcomes in this patient population are highly predictive of long-term recurrence risk. Using 3-year data from the EU Barricaid Controls, we developed a Cox proportional hazards model that included 15 variables including age, sex, body mass index, index level, smoking history, symptomatic reherniation by 3 months, back pain at baseline, back pain at 3 months, 3-month change in back pain, leg pain at baseline, leg pain at 3 months, 3-month change in leg pain, ODI at baseline, ODI at 3 months, and 3-month change in ODI. In multivariate analysis, symptomatic reherniation by 3 months and back pain severity at 3 months were independently associated with symptomatic reherniation risk at 3 years. Patients who were free from reherniation and with back pain scores under 13/100 at 3-month follow-up had a 3-year reherniation risk of 6.1%. In contrast, patients with early reherniation or 3-month back pain scores of 13/100 or higher had a 3-year reherniation risk of 22.6%. Additionally, the 1-year follow-up duration is clinically justified; among patients with a symptomatic reherniation through 3 years following Barricaid treatment, 60% were identified within the first year.^[26]^ Lastly, clinical outcomes during the first year after surgery are important metrics utilized by healthcare payers for making coverage decisions on new medical technologies. Another potential criticism of this study is a sample size of only 75 patients. This sample size was determined by calculating the minimum number of patients that would provide sufficient precision around the primary endpoint estimate. While formal noninferiority testing with patients in the US Barricaid Trial and the EU Barricaid Controls would have been preferable, such a study would be impractical since a sample size of over 1000 patients would be required to provide sufficient statistical power.

The importance of this trial primarily relates to describing the clinical performance of the Barricaid device in a US population with large post-discectomy annular defects, which has not been previously reported. Recurrent symptomatic herniation is associated with poor clinical outcomes and requires a technically demanding, expensive reoperation in most cases.^[11]^ With almost half a million lumbar discectomies performed in the US per year,^[27]^ and approximately 100,000 to 150,000 of these patients at high risk for recurrence based on annular defect size,^[12]^ this poses a widespread, clinically important problem not only for affected individuals but also for physicians, hospitals, and healthcare payers. Several studies have reported cost savings following Barricaid device implantation. Parker et al^[28]^ reported that use of the Barricaid device resulted in a $2200 savings per person compared with lumbar discectomy alone. Ament et al^[29]^ derived similar conclusions in which lumbar discectomy was $2100 less expensive when the Barricaid device was utilized. Should the rates of symptomatic recurrence in the current study approximate those observed in the EU Barricaid Controls, this would provide sufficient evidence that the Barricaid device is effective in reherniation prevention not only in European patients, but also in a US population of high-risk lumbar discectomy patients. The results of this study will have important clinical and economic implications for all stakeholders involved in treating patients with lumbar discectomy in the US.

## Acknowledgments

The authors had no writing assistance in the preparation of this manuscript.

## Author contributions

**Conceptualization:** K. Brandon Strenge, Christian P. DiPaola, Larry E. Miller, Clint P. Hill, Robert G. Whitmore.

**Formal analysis:** Larry E. Miller.

**Investigation:** K. Brandon Strenge, Christian P. DiPaola, Larry E. Miller, Clint P. Hill, Robert G. Whitmore.

**Methodology:** Larry E. Miller.

**Project administration:** K. Brandon Strenge, Christian P. DiPaola, Clint P. Hill, Robert G. Whitmore.

**Conceived and designed the study:** K. Brandon Strenge, Christian P. DiPaola, Larry E. Miller, Clint P. Hill, Robert G. Whitmore.

**Supervision:** K. Brandon Strenge, Christian P. DiPaola, Clint P. Hill, Robert G. Whitmore.

**Drafted the study protocol:** Larry E. Miller.

**Final approval of the version to be published:** K. Brandon Strenge, Christian P. DiPaola, Larry E. Miller, Clint P. Hill, Robert G. Whitmore.

**Responsible for data collection, analysis, and interpretation:** Larry E. Miller.

**Responsible for study implementation:** K. Brandon Strenge, Christian P. DiPaola, Clint P. Hill, Robert G. Whitmore.

**Reviewed and revised the study protocol:** K. Brandon Strenge, Christian P. DiPaola, Larry E. Miller, Clint P. Hill, Robert G. Whitmore.

**Writing – original draft:** Larry E. Miller.

**Writing – review & editing:** K. Brandon Strenge, Christian P. DiPaola, Clint P. Hill, Robert G. Whitmore.

Larry E. Miller orcid: 0000-0003-1594-1885.
